# A comparative population-based study of prostate cancer incidence and mortality rates in Singapore, Sweden and Geneva, Switzerland from 1973 to 2006

**DOI:** 10.1186/1471-2407-12-222

**Published:** 2012-06-06

**Authors:** Cynthia Chen, Nasheen Naidoo, Qian Yang, Mikael Hartman, Helena M Verkooijen, En Yun Loy, Christine Bouchardy, Kee Seng Chia, Sin Eng Chia

**Affiliations:** 1 Saw Swee Hock School of Public Health, National University of Singapore, 28 Medical Drive, Singapore, 11745, Singapore; 2 Department of Surgery, National University Hospital,Yong Loo Lin School of Medicine, 16 Medical Drive, Singapore, 117597, Singapore; 3 Imaging Division, University Medical Center Utrecht, Utrecht, The Netherlands; 4 Health Promotion Board, Ministry of Health, 3 Second Hospital Avenue, Singapore, 168937, Singapore; 5 Geneva Cancer Registry, Institute for Social and Preventive Medicine, University of Geneva, Geneva, Switzerland

## Abstract

**Background:**

Prostate cancer is the most commonly diagnosed malignancy in men in Sweden and Geneva, and the third most common in men in Singapore. This population-based study describes trends in the incidence and mortality rates of prostate cancer in Singapore, Sweden and Geneva (Switzerland) from 1973 to 2006 and explores possible explanations for these different trends.

**Methods:**

Data from patients diagnosed with prostate cancer were extracted from national cancer registries in Singapore (n = 5,172), Sweden (n = 188,783) and Geneva (n = 5,755) from 1973 to 2006. Trends of incidence and mortality were reported using the Poisson and negative binomial regression models. The age, period and birth-cohort were tested as predictors of incidence and mortality rates of prostate cancer.

**Results:**

Incidence rates of prostate cancer increased over all time periods for all three populations. Based on the age-period-cohort analysis, older age and later period of diagnosis were associated with a higher incidence of prostate cancer, whereas older age and earlier period were associated with higher mortality rates for prostate cancer in all three countries.

**Conclusions:**

This study demonstrated an overall increase in incidence rates and decrease in mortality rates in Singapore, Sweden and Geneva. Both incidence and mortality rates were much lower in Singapore. The period effect is a stronger predictor of incidence and mortality of prostate cancer than the birth-cohort effect.

## Background

Prostate cancer is the most commonly diagnosed malignancy in men in Sweden [1] and Geneva [2], and the third most common in men in Singapore [3]. Prostate cancer rates in Asian men in countries such as China [4] and Japan [5] are substantially lower than in Caucasians, however, the sharpest increase in prostate cancer incidence has most recently been observed in Asian countries [6], for example, incidence rates in Singapore have quadrupled over the past decade [3]. In Europe and the US, prostate cancer incidence rates have also increased and are most likely due to an increased awareness of prostate-related symptoms, improved access to health care, and increased diagnostic activity through prostate-specific antigen (PSA) testing [7].

**Table 1:** Age-adjusted incidence and mortality rates of all prostate cancers from 1973 to 2006, aged 20 and above for Singapore, Sweden and Geneva

**Country**	**Period**	**Incidence rate**		**Mortality rate**	
		**(per 100,000 person years)**		**(per 100,000 person years)**	
		**Total**	**Rate**	**Total**	**Rate**
Singapore	1973-1977	144	5.1	35	2.0
	1978-1982	240	6.8	69	3.4
	1983-1987	356	8.0	106	4.1
	1988-1992	529	9.6	187	5.7
	1993-1997	903	13.9	303	7.7
	1998-2002	1357	17.6	434	9.3
	2003-2006	1643	23.2	443	6.1
Sweden	1973-1977	17359	44.9	8516	21.6
	1978-1982	19202	46.0	8400	19.5
	1983-1987	22372	50.6	8795	18.9
	1988-1992	25329	55.4	10341	20.9
	1993-1997	28985	63.0	11411	21.6
	1998-2002	37313	84.5	12287	21.4
	2003-2006	38223	109.2	7625*	21.2*
Geneva	1973-1977	407	36.5	249	22.2
	1978-1982	524	42.1	270	21.1
	1983-1987	585	43.1	289	19.9
	1988-1992	724	50.0	317	19.7
	1993-1997	955	63.9	316	18.2
	1998-2002	1345	86.4	279	14.5
	2003-2006	1215	89.3	253	14.4

* Period for Sweden prostate mortality 2003–2005.

**Table 2:** Goodness of fit and likelihood ratio test for incidence rates in Singapore, Sweden and Geneva

**Goodness of fit**												
	**Singapore**				**Sweden**				**Geneva**			
	**(Negative binomial)**				**(Negative binomial)**				**(Negative binomial)**			
**Model**	Res Dev^*^	**Df**^**#**^	**AIC**^§^	**P-value**	**Res Dev**	**DF**	**AIC**	**P-value**	**Res Dev**	**DF**	**AIC**	**P-value**
**Age (A)**	31.5	42	473	0.882	24.4	42	817	0.632	38.3	42	500	0.986
**Age-Drift (AD)**	7.2	41	451	0.999	6.6	41	802	0.999	14.6	41	478	0.999
**Age-Period (AP)**	5.6	36	460	0.999	3.7	36	809	0.999	12.1	36	485	0.999
**Age-Cohort (AC)**	6.4	30	472	0.999	2.9	30	820	0.999	6.1	30	491	0.999
**Full APC**	5.0	25	481	0.999	2.0	25	829	0.999	5.1	25	500	0.999
**Likelihood ratio test**												
**Model**	**Dev**	**DF**	**P-value**		**Dev**	**DF**	**P-value**		**Dev**	**DF**	**P-value**	
**(H**_**0**_**vs H**_**1**_**)**												
**AP vs APC**	0.6	11	0.999		1.7	11	0.999		7.0	11	0.800	
**AC vs APC**	1.4	5	0.925		0.9	5	0.972		1.0	5	0.959	

^*^ Residual deviance.

^#^ Degree of freedom.

^§^ Akaike information criterion.

**Table 3:** Goodness of fit and likelihood ratio test for mortality rates in Singapore, Sweden and Geneva

**Goodness of fit**												
	**Singapore**				**Sweden**				**Geneva**			
	**(Negative binomial)**				**(Negative binomial)**				**(Poisson)**			
**Model**	Res Dev^*^	**Df**^**#**^	**AIC**^§^	**P-value**	**Res Dev**	**DF**	**AIC**	**P-value**	**Res Dev**	**DF**	**AIC**	**P-value**
**Age (A)**	13.5	42	402	0.999	1.1079	42	641	0.999	72.0	42	282	0.003
**Age-Drift (AD)**	13.3	41	404	0.999	1.1077	41	643	0.999	40.4	41	252	0.495
**Age-Period (AP)**	8.7	36	409	0.999	0.71	36	653	0.999	32.4	36	254	0.642
**Age-Cohort (AC)**	6.9	30	419	0.999	0.50	30	665	0.999	34.1	35	263	0.512
**Full APC**	5.0	25	428	0.999	0.28	25	674	0.999	27.6	30	267	0.594
**Likelihood ratio test**												
**Model (H**_**0**_**vs H**_**1**_**)**	**Dev**	**DF**	**P-value**		**Dev**	**DF**	**P-value**		**Dev**	**DF**	**P-value**	
**AP vs APC**	3.7	11	0.979		0.43	11	0.999		4.8	6	0.570	
**AC vs APC**	1.9	5	0.869		0.22	5	0.999		6.5	5	0.258	

^*^ Residual deviance.

^#^ Degree of freedom.

^§^ Akaike information criterion.

PSA testing received US Food and Drug Administration approval as a monitor for treatment response in 1986 and was subsequently approved as a screening aid for diagnosis in 1994 [8]. PSA testing has subsequently contributed to a doubling in the incidence rates of prostate cancer in the US between 1986 and 1992 [9]. Similarly, after PSA screening became available in the Nordic countries circa. 1990, rapid increases in PSA testing were also shown to be associated with sharp increases in prostate cancer incidence [10].

Despite increasing incidence rates globally, mortality rates of prostate cancer have declined in several countries [11,12]. A comparison of the incidence and mortality rates between Singapore and European countries, such as Sweden and Geneva, could reveal important insights into these trends. Thus, our study aimed to compare the incidence and mortality rates of prostate cancer in Singapore, Sweden and Geneva.

## Methods

### Data source

#### Incidence and mortality data (1973–2006)

##### Singapore

Comprehensive population-based cancer registration in Singapore began in January 1968, with the aim of providing current information on cancer patterns and trends. Data was obtained from different sources, namely notifications by physicians, pathology records, hospital records, and death certificates. Population based data for incidence and mortality rates for Singapore was taken from the Singapore Cancer Registry, National Registry of Diseases Office (NRDO) (n = 5,172). The denominators for incidence and mortality were the total number of person-years from the Singapore resident population based on the Singapore Population Census 2000 updated reports [13].

##### Sweden

Sweden was selected as a country of comparison as it has one of the highest incidence rates of prostate cancer globally [14]. Incidence and mortality population based data for Sweden was extracted from the NORDCAN database (http://www-dep.iarc.fr /nordcan/English/frame.asp) (n = 188,783). This database contains data on the prevalence, incidence and mortality of 41 cancers, including prostate cancer, from each Nordic countries’ national cancer and mortality registries over the past 60 years [15] and which is updated approximately twice a year. Coverage of incident cases in each registry was previously reported to be close to 100% [16,17]. Data was collected from multiple sources, including physicians, hospitals, institutions with hospital beds, pathological and cytological laboratories, and by linkages with administrative health/disease registers. Our study contained incidence and mortality data extracted from the NORDCAN database over the period 1973 to 2006.

##### Geneva

Geneva was selected for comparison due the availability of a complete dataset for analysis. Population based data on incidence and mortality for Geneva was used over the period 1973 to 2006. Data on incident cases and deaths were obtained from the Geneva Cancer Registry (Institute for Social and Preventive Medicine, Geneva University, Geneva, Switzerland) and the ‘Office Cantonal de la Population’ respectively (n = 5,755). The Geneva Cancer Registry recorded all incident cancers occurring in the population of the canton (approximately 465,000 inhabitants in 2007) since 1970. Information was collected from various sources [18] and is considered accurate with <2% of cases recorded from death certificates only [19,20]. Trained tumor registrars systematically extracted data from medical and laboratory records. Cause of death was established from clinical files and/or information from physicians.

### Statistical analysis

Incidence rates and mortality rates were defined as the number of new cases and the number of deaths, respectively, in a given period for a specific population. To adjust for the differences in the age structure and to account for the strong influence of age on the risk of cancer, the incidence rates and mortality rates were age-standardised to the world standard million population and expressed as 100,000 person years for persons aged 45 years and above. Data were analysed using five year age groups (45–49, 50–54, …70-74, 75+ years) for all three countries. As those aged 45 years and above constituted only 26% in the world standard million population, we recalibrated the weighting to 100% based on the respective weights of each age group.

The number of incident cases and deaths were assumed to follow a Poisson distribution and hence we used log-linear Poisson modeling of cases based on age and year of diagnosis (period) and year of birth (birth-cohort). When over-dispersion (variance greater than the mean) was detected while modeling the incidence or mortality, a negative binomial distribution was used [21] instead of the original Poisson distribution, as a negative binomial distribution has a larger variance than a Poisson distribution.

A limitation of using only age-standardised rates to describe the trends in incidence and mortality rates is that this does not account for period and birth-cohort effects. Use of the age-period (AP) and age-birth-cohort (AC) models was applied to disentangle the separate effects of period of diagnosis and birth-cohort on incidence and mortality. Period effects tend to influence all individuals simultaneously during a particular time period regardless of their age, whilst birth-cohort effects are attributable to certain factors related to the birth year. The analysis was based on the generalized linear model approach. We also considered the scenario where the effects of period and birth-cohort in AP and AC models, respectively, were assumed to be linear and hence inseparable. In such cases, the combined linear model is referred to as the age-drift model [22]. It is well established that there is a linear dependency between age, period and birth-cohort as the birth cohort can be computed by subtracting age at diagnosis from the calendar period of diagnosis.

The deviance statistic was used to assess the goodness of fit of the models. A non-significant p-value (> 0.05) indicated a good fit. Difference in deviance and the likelihood ratio test were used to compare different models, where a significant p-value indicates that the more complicated model has significant improvement over the simpler model. The deviance and change in deviance due to cohort or period effects were compared to the age-period-cohort (APC) model. The cohort effect was tested in comparison to the AP and APC models and likewise, the period effect was tested in comparison to the AC and APC models.

The Akaike Information Criterion (AIC) was also calculated to compare models with different complexities that are not required to be nested within each other, with smaller AIC values suggestive of a better model fit [23]. We compared both the AP and AC models with the APC model and selected the most parsimonious model based on the likelihood ratio test and AIC criteria.

Ethical approval for this study was granted by the National University of Singapore IRB (approval number: NUS-747).

## Results

### Incidence

**Figure 1:**
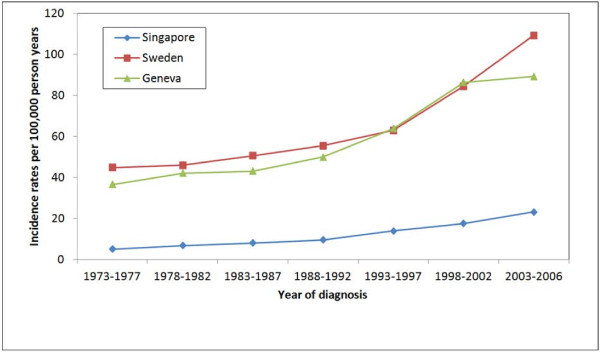
Age-standardised (world standard million population) prostate cancer incidence rates per 5-year period (1973–2006) stratified by country for Singapore, Sweden and Geneva.

Overall, incidence rates increased during the period 1973 to 2006 (Figure 1, Table 1). Singapore had the lowest age-standardised incidence rates for all 5-year periods while Sweden had the highest, with the exception of the period 1993 to 2002. All three countries had relatively sharper rises in incidence rates in the subsequent periods after 1993 to 1997, more so for Sweden and Geneva compared to Singapore.

**Figure 2:**
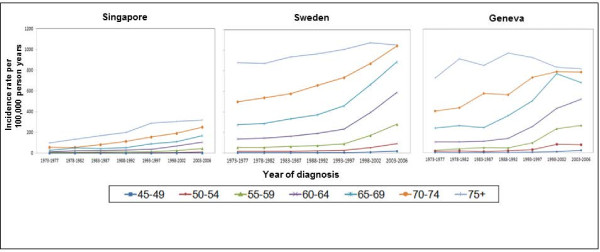
Age-specific prostate cancer incidence rates (per 100,000 person-years) per 5-year period stratified by 5-year age group for Singapore, Sweden and Geneva.

Higher age-specific incidence rates were found in older age-groups and increased in the later years of diagnosis for all three countries (Figure 2). This increase was more prominent in the age groups 55 to 74 years, with sharper rises compared to younger age groups. In the age group 75 years and above, the rate of incidence increase was less steep in the period after 1993 for both Singapore and Sweden, despite increases in the overall age-specific incidence rates. There was a decrease in incidence rates for Geneva in the oldest age group since 1993. The age-specific incidence rate overall for Geneva and Sweden was approximately three times higher than that of Singapore.

### Mortality

**Figure 3:**
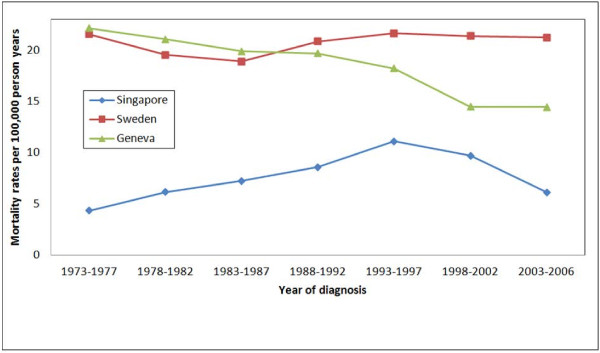
Age-standardised (world standard million population) prostate cancer mortality rates per 5-year period (1973–2006) stratified by country for Singapore, Sweden and Geneva.

Overall, Singapore had the lowest age-standardised mortality rates over all periods measured (Figure 3, Table 1). Both Geneva and Singapore experienced drops in mortality rates from 1993 to 2006 whereas the mortality rate for Sweden remained relatively constant (Figure 3). Geneva had higher mortality rates as compared to Sweden from 1973 to 1987, with a sharp decline thereafter. Singapore mortality rates showed a steady rise from 1973 to 1997 and a sharp decline in the subsequent two periods.

**Figure 4:**
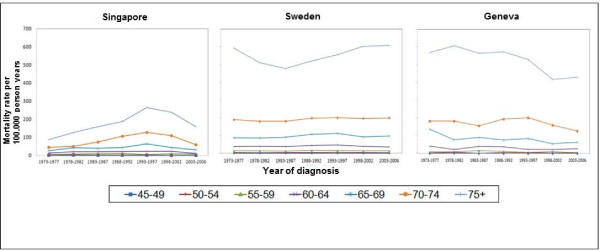
Age-specific prostate cancer mortality rates per 5-year (per 100,000 person-years) period stratified by 5-year age group.

Higher age-specific mortality rates were found in the older age groups for all three countries (Figure 4). In both Singapore and Geneva, there was a drop in mortality rates for the age group 65 years and above from 1993 to 2006 (Figure 4). In Sweden, the age-specific mortality rates remained unchanged since 1973 for those aged between 45 and 74 years. The oldest age group (75+ years) had mortality rates following a U-shaped pattern.

### APC modeling

The negative binomial distribution was preferred based on the goodness of fit test when modeling incidence in all three countries and mortality for Singapore and Sweden. The Poisson distribution was preferred when modeling for mortality for Geneva. The likelihood ratio test for incidence rates in all countries indicated that both the AP model and the AC models were sufficient in explaining the variation in the full APC model for Singapore (P-value = 0.999 (AP vs APC), 0.925 (AC vs APC)), Sweden (P-value = 0.999, 0.975) and Geneva (P-value = 0.800, 0.959) (Table 2).

Similarly, the likelihood ratio test for mortality rates indicated that both the AP model and AC model were also sufficient in explaining the variation in the full APC model for Singapore (P-value = 0.979, 0.869), Sweden (P-value = 0.999, 0.999) and Geneva (P-value = 0.570, 0.258) (Table 3).

## Discussion

In general, the age-standardised incidence rates of prostate cancer above the age of 50 years in all three countries increased between 1973 and 2006, and occurred at a faster rate in Sweden and Geneva than in Singapore. The sharper rise occurring in Sweden after the early 1990s is consistent with the increasing availability of PSA testing [24] and may also be attributed to the more frequent use of transurethral resection of the prostate and invasive diagnostic procedures such as random biopsies [25]. In Singapore, PSA screening is currently recommended for males aged 50 to 75 years and with a first degree relative diagnosed before age 65 years [26], whereas approximately 1 in 2 males above age 50 years in Sweden (56 % in 2007 [27]) and Geneva (55 % in 2005 [28]) had PSA screening. This could explain the sharper rises in incidence rates after 1993 in Sweden and Switzerland compared to Singapore. It is possible that the continuous rise in incidence rates in Singapore may represent a real increment of incident cases, given the highly selective criteria for screening. A further potential risk factor for these increasing incidence rates in Singapore could be due to the adoption of a more Westernized diet that generally has a higher intake of animal fats [29]. In comparison, soy based Asian diets have been shown to provide some protection against prostate cancer [30,31].

As the upper limit for screening in Singapore [26], Sweden [27] and Geneva [32] is up to 75 years, it is expected that the rate of increase in incidence in the 75+ year age group is slower (Figure 2). The higher overall age-specific incidence rates for Geneva and Sweden may be explained by the higher baseline incidence and higher screening rates [28]. In addition, rapidly aging populations in the three countries will have a growing number of men reaching an older age which may contribute to the disease becoming more frequently diagnosed [33].

The age-standardised mortality rates declined in the later periods for all three countries. From our results, mortality rates for Geneva declined steadily from 1973 onwards whereas for Singapore there was a steady rise in mortality rates from 1968 to 1992 with a recent decline from 1993 onwards. This raises the question of whether this could be an effect of PSA testing. To date there is little conclusive evidence that PSA-based screening reduces prostate cancer mortality. However, recent randomised controlled trials have shown contradicting results. The Prostate, Lung, Colorectal, and Ovarian (PLCO) Cancer Screening Trial in the US showed a marginal increase in the incidence and concluded that there was no mortality reduction with combined PSA and digital rectal examination screening over an 11 year median follow-up [34]. The UK based European Randomised Study of Screening for Prostate Cancer (ERSPC) trial after a median follow-up of 11 years showed an increased incidence and a 21% relative reduction in risk of death that was only marginally statistically significant at p = 0.001 [35].

Treatment of prostate cancer does not differ greatly between the three countries yet the mortality rate is declining much faster in Singapore and Geneva compared to Sweden. It is uncertain whether this observation can be explained by genetic differences in the populations, different environmental factors or a combination of both (gene-environment interactions) [36]. Alternatively, it may argued that the drop in mortality is due to technological advancements in diagnosis and treatment of the disease, such as newer surgical approaches for localized disease, improved irradiation techniques and hormonal/antiandrogenic therapy [11], or due to a combination of all these factors. The relatively sharper drop in mortality rate in Geneva may be due to the adoption of hormonal therapy [18].

From the APC modeling, the incidence and mortality rates of prostate cancer appeared to be more strongly associated with the age and period effect than with the birth-cohort effect in all three countries, based on the lower calculated AIC criteria and likelihood ratio test results. Older males tended to be at higher risk of developing prostate cancer and in the later periods. In addition, mortality was higher in older males and across the earlier periods, which could be confounded by PSA testing. Males of older age and in the later period of diagnosis had a higher incidence, whereas males of older age and in the earlier period had higher mortality rates for prostate cancer.

A limitation of this study was that it extended over a relatively long time period during which changes in diet, environmental and diagnostic factors are likely to have occurred. It would be a further challenge to identify the independent factors influencing the change in the trends of incidence and mortality as it could be due to individual factors acting independently or in combination. Age-standardized rates for incidence and mortality in our study are not comparable with other published data as different weightings were used. Accurate interpretation of the incidence and mortality trends in the three countries would be incomplete without data on PSA screening as a potential confounder. As data on individual PSA testing was not available, it was not possible to separate the effect of the real increment in incident cases from over-diagnosis due to increased screening, as we would need to establish whether patients had PSA screening prior to diagnosis.

## Conclusion

Our analysis showed that overall age-standardised incidence rates of prostate cancer increased over the period 1973 to 2006 and that the mortality rates declined over the later period (1998 to 2006) in all three countries. Both incidence and mortality rates were much lower in Singapore than in Sweden and Geneva. The mortality rates for Singapore followed an inverted U-shape whereas the mortality rates for Sweden remained relatively unchanged and Geneva experienced a steady decline over the period 1973 to 2006. Based on APC modeling, the age and period effects were shown to be more strongly associated with incidence and mortality than the birth cohort effect.

## Competing interests

The authors declare that they have no competing interests.

## Authors’ contributions

CC carried out all statistical analysis and drafted the manuscript. NN and QY were involved in drafting and critical review of the manuscript. MH was involved in drafting the manuscript and provided the Swedish population data. HMV drafted the manuscript and provided the population data from Geneva. EYL provided the Singapore population data. CB provided the Geneva population data and was involved in manuscript writing. KSC conceived the study and provided critical review of the manuscript. SEC conceived the study, and participated in the study design and coordination and helped to draft the manuscript. All authors read and approved the final manuscript.

## Pre-publication history

The pre-publication history for this paper can be accessed here:

http://www.biomedcentral.com/1471-2407/12/222/prepub
